# The evolution to hepta-refractory myeloma involves sequential loss of CD38, BCMA and GPRC5D

**DOI:** 10.1038/s41375-026-02889-3

**Published:** 2026-02-17

**Authors:** C. Riedhammer, M. Truger, H. Lee, LB Leypoldt, M. Meggendorfer, S. Hutter, H. Müller, J. Mersi, SK Kadel, T. Buchwald, R. Kosch, M. Helal, N. Afrin, A. Rosenwald, E. Gerhard-Hartmann, A. Brioli, J. Krönke, T. Haferlach, C. Haferlach, KC Weisel, P. Neri, H. Einsele, KM Kortüm, JM Waldschmidt, N. Bahlis, N. Weinhold, L. Rasche

**Affiliations:** 1 Department of Internal Medicine 2, University Hospital of Würzburg, Würzburg, Germany; 2 MLL Munich Leukemia Laboratory, Munich, Germany; 3 Arnie Charbonneau Cancer Institute, University of Calgary, Calgary, AB Canada; 4 Department of Hematology, Oncology and Bone Marrow Transplantation With Section of Pneumology, University Medical Center Hamburg-Eppendorf, Hamburg, Germany; 5 Mildred Scheel Early Career Center, University Medical Center Hamburg-Eppendorf, Hamburg, Germany; 6 Mildred Scheel Early Career Center, University Hospital Würzburg, Würzburg, Germany; 7 Institute of Pathology, University of Würzburg, Würzburg, Germany; 8 Department of Internal Medicine C, University Hospital of Greifswald, Greifswald, Germany; 9 Hannover Medical School, Clinic for Hematology, Hemostaseology, Oncology and Stem Cell Transplantation, Hannover, Germany; 10 Heidelberg Myeloma Center, Department of Medicine V, University Hospital Heidelberg, Medical Faculty, Heidelberg University, Heidelberg, Germany; 11 Clinical Cooperation Unit Molecular Hematology/Oncology, Department of Internal Medicine V, Heidelberg University Hospital, Heidelberg University and German Cancer Research Center (DKFZ), Heidelberg, Germany

**Keywords:** Myeloma, Translational research

## Abstract

Multiple myeloma (MM) resistant to CD38 antibodies, two immunomodulatory drugs (IMiDs), two proteasome inhibitors (PIs), and both BCMA- and GPRC5D-directed immunotherapies defines hepta-refractory MM, a novel end-stage entity. In a multi-center cohort of 37 patients, median overall survival was 12.8 months, with progression-free survival across salvage therapy lines of only 2.7–3.7 months. Whole genome sequencing (WGS) revealed frequent biallelic tumor suppressor gene events, particularly *TP53*, consistent with proliferative, apoptosis-resistant disease. Genomic alterations linked to IMiD, BCMA, GPRC5D, and CD38 resistance occurred in 71%, 41%, 35%, and 12% of patients, respectively. Almost one-third of patients showed concurrent loss of BCMA (*TNFRSF17*) and GPRC5D. Sequential WGS demonstrated branching evolutionary trajectories with multiple distinct *TNFRSF17* and *GPRC5D* variants arising within individual patients, pointing to a hidden reservoir of persistent clones with ongoing mutational processes even after deep remissions. Immunohistochemistry (IHC) confirmed loss of BCMA expression caused by biallelic *TNFRSF17* genomic events but also revealed loss of expression attributable to other mechanisms. Importantly, BCMA status predicted benefit from BCMA re-treatment. Hepta-refractory MM is marked by profound genomic complexity, antigen loss, and poor outcomes, highlighting the need for novel therapies and broader diagnostics such as integrated genomic and IHC testing for this ultra-refractory population.

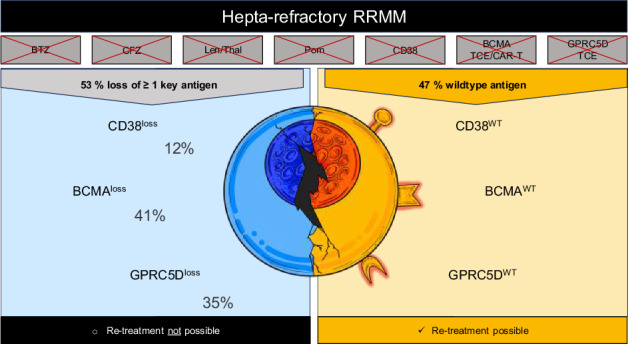

## Introduction

No other hematologic malignancy has seen more drug approvals in recent decades than multiple myeloma (MM), including some of the most advanced new drug classes. Yet, the disease remains largely incurable. Patients who have been refractory to two immunomodulatory drugs (IMiDs), two proteasome inhibitors (PIs), and a CD38 antibody, referred to as “penta-refractory”, used to face a median survival of only 5.6 months [1]. The advent of novel immunotherapies, such as CAR T-cells and bispecific T-cell-engaging antibodies (TCE) targeting plasma cell antigens like BCMA and GPRC5D, has markedly improved treatment outcomes for this population. However, even these new immunotherapies have not yet been able to induce a survival plateau, and most patients inevitably relapse [2–8]. These “hepta-refractory” patients (penta-refractory plus resistance to BCMA- and GPRC5D-targeting therapies) are increasingly seen in clinical practice. Understanding the mechanisms driving resistance and relapse is key to guiding treatment strategies for these patients.

MM is a disease with profound subclonal heterogeneity and branching evolutionary pathways, making treatment responses difficult to predict [9, 10]. The concept of “clonal tidings” provided an explanation for why patients may respond to therapies to which they were previously resistant [11, 12]. At the same time, inactivating or structurally modifying mutations in tumor suppressor genes or in genes encoding drug targets clearly accumulate in relapsed and refractory patients [13, 14]. This observation is particularly true for relapse following T-cell targeting therapies and, to some extent, also for antibody-drug conjugates. Several studies have shown that immunotherapies can act as an evolutionary bottleneck, selecting for clones with antigen loss due to homozygous target gene deletions or point mutations leading to “functional” loss [15–20]. Therefore, in the context of hepta-refractory disease, it will be crucial to determine whether these aberrations persist in subsequent relapses and whether they accumulate when choosing different targets, especially since diagnostic testing for antigen expression is, in principle, possible, and could guide treatment decisions.

In this multicenter study, we investigated hepta-refractory disease using whole genome sequencing (WGS) together with immunohistochemistry (IHC) in a unique MM population that has exhausted all available treatments. We demonstrate that prior therapies leave distinct genomic traces in MM cells, including the stepwise loss of BCMA and GPRC5D. However, in patients with intact target expression, retreatment with CAR T-cells remained a viable option.

## Methods

All work was approved by the respective health research ethics boards (EK 8/21, HREBA.CC-21-0248). Written consent for the sample collection and analyses was obtained in accordance with the Declaration of Helsinki.

### Clinical data analysis

Records of 37 patients treated at the University Hospitals of Würzburg, Hamburg-Eppendorf, Greifswald and the University of Calgary were analyzed retrospectively. Treatment response and number of prior lines of therapy (LOT) were determined according to the International Myeloma Working Group criteria [21] and international consensus [22]. The definition of genetic high-risk included del(17p), *TP53* mutation, biallelic del(1p32) and combinations of *IGH* translocations t(4;14), t(14;16), and t(14;20), del(1p32) and 1q21 gain/amplification [23]. Time-to-event analyses were performed in R (version 2023.09) using Kaplan-Meier methods.

### Whole genome sequencing

All patients provided written informed consent for WGS prior to sample collection. Bone marrow aspirates, specimens from extramedullary sites and cytological material were collected from patients as part of routine clinical procedures. For samples collected from patients of the University Hospitals of Würzburg, Hamburg-Eppendorf and Greifswald, WGS was performed at the Munich Leukemia Laboratory (MLL).

WGS libraries were prepared from 1 μg of DNA from CD138+ purified cells with the TruSeq PCR-free library prep kit, and 2 × 150-bp paired-end sequences were generated on a NovaSeq 6000 instrument (Illumina) with a median coverage of 107x. A tumor/matched normal workflow or, in cases with no sample-specific normal tissue available, a tumor/unmatched normal workflow was used for variant calling. Reads were aligned to the human reference genome (GRCh37) using Isaac aligner (v.03.16.02.19) through the BaseSpace WGS app v.5 (Illumina) with default parameters. SNVs and small indels (<50 bp) were called with Strelka Somatic Variant Caller (v.2.4.7) and SVs with Manta (v.0.28.0). Each variant with a PASS flag was queried against the gnomAD database (v.2.1.1), and variants with global population frequencies >0.05% were excluded to reduce germline calls. Further analysis was performed on protein-altering and splice-site variants only. SNVs in 135 genes of interest (Supplementary Table 1) were analyzed in detail. CNVs were called using GATK4 (v.4.0.8.1, Broad Institute). Copy neutral loss of heterozygosity (CN-LOH) was assessed using HadoopCNV.

Patients MM#16 and MM#17 of the University of Calgary were included in a previously published study (as MM-03 and MM-19), and WGS on isolated CD138+ cells was performed at the New York Genome Center as reported [16]. Libraries were prepared using the TruSeq DNA Nano Library Preparation Kit (Illumina). Briefly, 100 ng DNA was sheared using a Covaris LE220 sonicator. DNA fragments underwent bead-based size selection, were end-repaired, adenylated, ligated to Illumina sequencing adapters and amplified. Final libraries were sequenced on a Nova-Seq 6000 sequencer (Illumina) using 2 × 150-bp cycles. Variant calling and interpretation were performed at the MLL as described above.

### Analysis of mutational signatures

Mutational signatures were identified de novo and matched to Cosmic mutational signatures v.3.1 (http://cancer.sanger.ac.uk/cosmic/signatures) using mutSignatures (version 2.1.1) [24]. The relative contributions of these signatures for each sample were then re-estimated and plotted using the R package mmsig (v.0.0.0.9000) [25].

### Immunohistochemistry

Specimens were obtained by puncture from bone marrow, extramedullary sites or cytologic material according to standard procedures. Histological sections (2 μm) were cut and stained with hematoxylin and eosin and Giemsa for routine histological evaluation. IHC was performed on formalin-fixed paraffin-embedded tissue slides using an automated immunostainer (BOND-III, Leica Biosystems) according to the manufacturer’s instructions and standard protocols. The BCMA-antibody E6D7B, Cell Signaling, was used in a dilution of 1:100. In addition to descriptive evaluation, semi-quantitative analysis of IHC staining was performed using a multiplication score. Both membranous and dot-like cytoplasmic positivity (Golgi area) for BCMA was observed; however, only membranous staining was considered for the score.

### Phylogenetic reconstruction

Mutations from paired samples were analyzed with the R package MOBSTER (v1.0.0) to differentiate positively selected subclones from neutrally evolving tails [26]. Mutations classified as tail mutations in one sample but assigned to cluster C1 in the paired sample were retained. VIBER (v0.1.0) was used for cluster assignment, and clonal hierarchies were reconstructed with clonevol (v0.99.11). Phylogenetic trees were then manually curated, with branch lengths not representing the extent of subclonal divergence.

## Results

### Clinical characteristics and outcome of patients with hepta-refractory MM

**Fig. 1:**
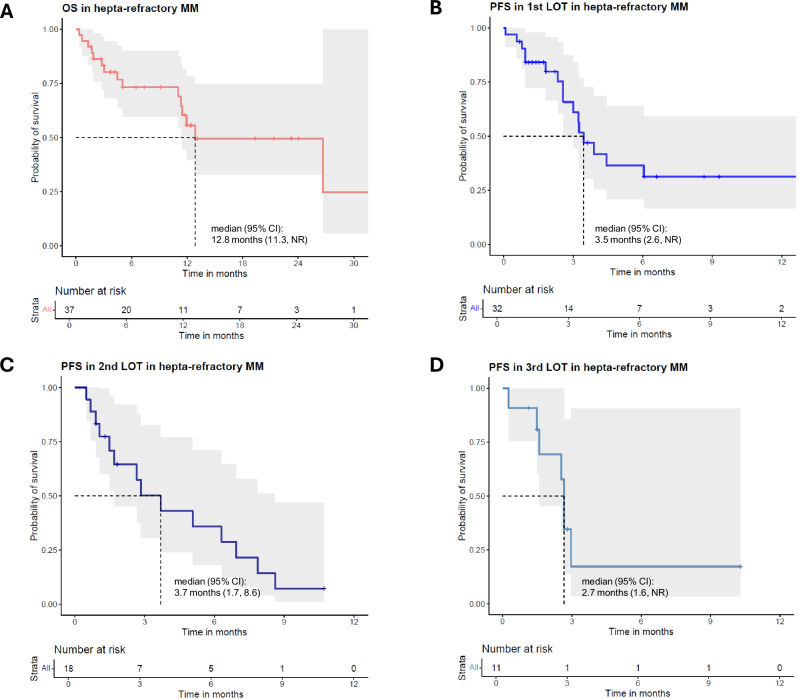
Outcomes of patients with hepta-refractory MM. Kaplan-Meier Curves for **A** OS **B** PFS in first LOT **C** PFS in second LOT **D** PFS in third LOT in hepta-refractory MM. *CI* confidence interval. Shading shows 95% confidence bands.

We enrolled 37 patients who met the criteria for hepta-refractory disease, adhering to the IMWG definition of resistance [21, 27]. The patients were heavily pretreated, with a median of 9 (range: 5–17) prior LOT and a median time from initial diagnosis of 7.6 years (range: 2.0–20.1). The majority of patients had extramedullary disease (62.5%), genetic high-risk (64.7%) according to the latest assessment, and a high tumor burden (median bone marrow infiltration of 50%, Supplementary Table 2). Median progression-free survival (PFS) in subsequent LOT after occurrence of hepta-refractoriness was 2.7–3.7 months, median overall survival (OS) was 12.8 months (Fig. 1). A detailed description of the additional LOT is provided in Supplementary Table 3. Although outcomes were poor, there was notable heterogeneity among patients, with some progressing rapidly while others benefited from salvage therapies, indicating distinct disease characteristics.

### Enrichment of single and double hits in tumor suppressor genes

To further investigate the disease characteristics, we performed WGS for a subset of the above-described cohort. In total, 21 WGS were available from 17 patients, including longitudinal samples from three individuals. For eight of these patients, the plasma cell content at the iliac crest was insufficient for WGS. Therefore, biopsies from extramedullary disease (EMD) were analyzed instead.

**Fig. 2:**
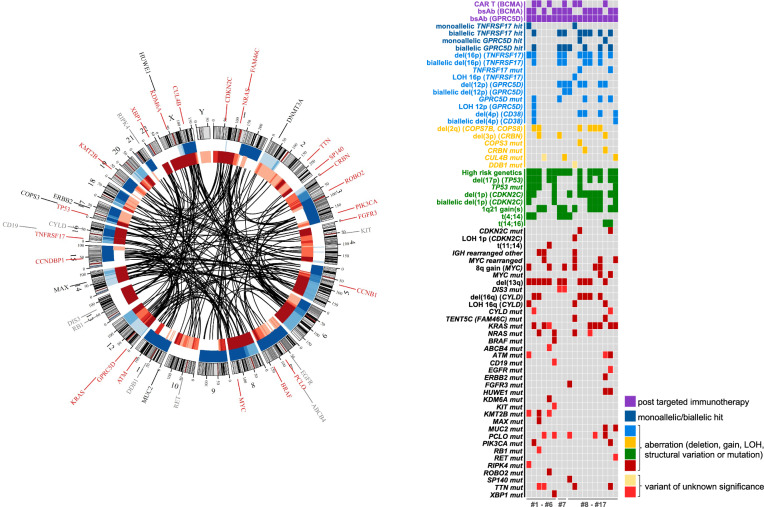
Merged circos plot and oncoplot of hepta-refractory MM patients MM#1 to MM#17. Circos plot: Black lines indicate structural variants between chromosomes. Blue bars indicate copy number gains, red bars indicate copy number losses. Genes with mutations (tier 1 in red, tier 2 in black) and variants of unknown significance (tier 3 in gray) are indicated according to their chromosomal localization in the outer circle. Oncoplot: Targeted immunotherapies (BCMA, GPRC5D; purple) and landscape of copy number variations (gains and deletions), structural variations (e.g. *IGH* translocations), loss of heterozygosity (LOH), mutations (tier 1 and tier 2) and variants of unknown significance (tier 3) in genes of interest based on WGS data. Genes/aberrations associated with targeted immunotherapy (blue) or with treatment with IMiDs (yellow), high risk genetic features (green) and additional alterations of interest (black) are listed. Of patient MM#7, the results of WGS analyses in hepta-refractory status from two different time points are included (2023 and 2025 after subsequent BCMA-directed CAR T-cell therapy).

WGS revealed a highly complex genomic landscape, comprising numerous chromosomal aberrations and single-nucleotide variants (SNVs) (Fig. 2 and Supplementary Table 4). All 17 patients carried at least one high-risk aberration, and according to the most recent IMS/IMWG consensus recommendations [23], 13 patients (77%) were classified as high-risk MM.

Among the lesions conferring enhanced clonal fitness and proliferative advantage, we identified del(17p) in 10 patients (59%), and biallelic *TP53* inactivation (del(17p) plus *TP53* mutation) in 6 (35%). Alterations of the tumor suppressor gene *CYLD* were present in 10 patients (59%), while *CDKN2C* aberrations were detected in 12 cases (71%), including 6 with biallelic deletion, which Schavgoulidze et al. recently characterized as “ultra-high risk myeloma” [28]. Notably, all biallelic *TP53* lesions and 5 of 6 biallelic *CDKN2C* deletions were detected in EMD samples, consistent with aggressive biology. SNVs were also detected in the tumor suppressor gene *FAM46C* in 2 patients (12%), and in the oncogenes *KRAS*, *NRAS*, and *BRAF* in 8 (47%), 4 (24%), and 1 patient, respectively (Fig. 2).

In summary, the genomes of hepta-refractory patients exhibit hallmarks of very advanced disease, characterized by pervasive single and double hits in tumor suppressor genes and oncogenes.

### Massive accumulation of drug resistance mutations

In the second step, we leveraged WGS data to assess the frequency and nature of genomic alterations associated with prior drug exposure. We found none of the aberrations previously associated with PI resistance, such as mutations in *PSMB5*, *PSMC2*, *PSMC6*, and *PSMD1* [14, 29–32]. In contrast, the CRBN pathway was frequently mutated (*n* = 12, 71%). *CRBN*, which encodes the IMiD-binding protein cereblon, was disrupted by deletions in 3 patients (18%); in addition, 2 patients carried *CRBN* mutations, one of whom also harbored a deletion and in a previous analysis a structural variant of chromosomes 1 and 3 resulting in a CEP350::CRBN fusion. SNVs were also identified in *CUL4B* (3 patients, 18%), *COPS3* (1 patient), and *DDB1* (1 patient). Deletions affecting 2q37, including *COPS7B* and *COPS8*, were observed in 6 patients (35%).

Focusing on targets of immunotherapies, *CD38*, essential for anti-CD38 monoclonal antibody activity, was impacted by 4p deletions or monosomy 4 in 5 patients (29%), including 2 with complete biallelic loss. *TNFRSF17* (encoding BCMA), the target of CAR T-cells and bispecific antibodies, was affected by monoallelic loss in 2 patients and double-hit genomic events in 7 (41%). 6/7 patients with biallelic *TNFRSF17* aberrations had previously undergone treatment with a BCMA-directed TCE. All patients had received GPRC5D-directed treatment with talquetamab. Biallelic *GPRC5D* aberrations were present in 6 patients (35%) and monoallelic loss in 2.

**Fig. 3:**
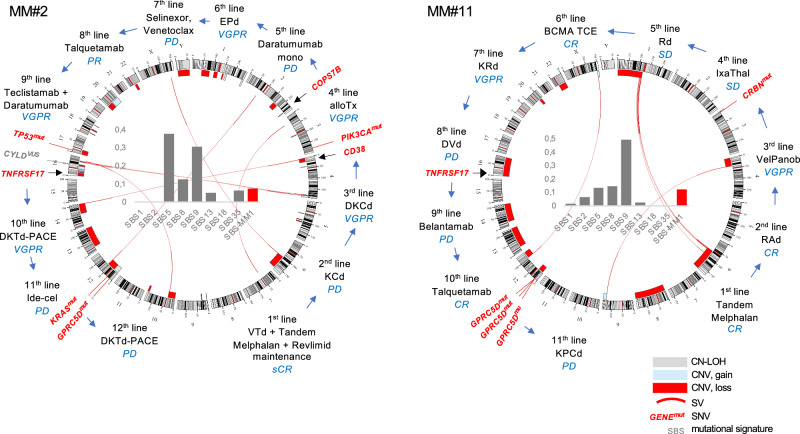
Circos plot of hepta-refractory patients MM#2 and MM#11. The bar chart inside indicates specific mutational signatures (SBS-MM1, associated with melphalan exposure in red). Red lines indicate structural variants between chromosomes. Blue and red bars indicate copy number gains and losses. Gray bars indicate copy-neutral loss of heterozygosity (CN-LOH). Genes with mutations (mut, tier 1 and 2 in red) and variants of unknown significance (VUS, tier 3 in gray), as well as chromosomal localization of genes of interest, are shown in the outer circle. Lines of therapy are shown on the outside. DVd daratumumab-bortezomib-dexamethasone, DKCd daratumumab-carfilzomib-cyclophosphamide-dexamethasone, DKTd-PACE daratumumab-carfilzomib-thalidomide-dexamethasone-cisplatin-doxorubicine-cyclophosphamide-etoposide, EPd elotuzumab-pomalidomide-dexamethasone, IxaThal ixazomib-thalidomide, K(P)Cd carfilzomib-(pomalidomide-)cyclophosphamide-dexamethasone, KRd carfilzomib-lenalidomide-dexamethasone, RAd lenalidomide-doxorubicin-dexamethasone, Rd lenalidomide-dexamethasone, VelPanob bortezomib-panobinostat.

For patients MM#2 and #11, WGS profiles closely reflected treatment history, effectively functioning as a “molecular medical record” (Fig. 3). In patient MM#2, the SBS-MM1 (SBS99) melphalan mutational signature accounted for 7.4% of mutations, in line with an ASCT during first-line therapy. This patient also carried a 2q37 deletion associated with IMiD resistance, biallelic deletions of *TNFRSF17* and *CD38,* as well as aberrations affecting both copies of *GPRC5D* (copy-neutral LOH of 12p with a *GPRC5D* mutation), severely limiting therapeutic options. Similarly, patient MM#11 exhibited multiple therapy-associated alterations: (i) SBS-MM1 signature (12.2% of mutations), (ii) a nonsense *CRBN* mutation (p.Gln8*), (iii) biallelic *TNFRSF17* deletion, and (iv) *GPRC5D* deletion plus three subclonal *GPRC5D* mutations.

Taken together, our data show that the genomic makeup of hepta-refractory disease is consistent with high-risk disease and relapse from intensive chemotherapy, but also includes heterogeneity caused by specific alterations related to prior use of IMiDs and immunotherapies.

### Detailed description of resistance mutations in BCMA and GPRC5D

Given the multiple BCMA- and GPRC5D-targeting agents currently in development, each with potentially distinct vulnerabilities to structural variants of these antigens [16], we analyzed biallelic aberrations of these two targets in more detail (Table 1).

**Table 1:** WGS analysis for *TNFRSF17* (BCMA) and *GPRC5D*.

ID	post CAR-T (BCMA)	post TCE (BCMA)	post TCE (GPRC5D)	*TNFRSF17*		*GPRC5D*		*TNFR SF17* deletion	*TNFRSF17* mutation				*TNFR SF17* LOH	*GPR C5D* deletion	*GPRC5D* mutation				*GPRC5D* LOH
				mono-allelic hit	bi- allelic hit	mono-allelic hit	bi-allelic hit		DNA/cDNA	Protein	Exon	VAF (%)			DNA/cDNA	Protein	Exon	VAF (%)	
MM#1	-	+	+	+	-	-	-	mono- allelic	-	-	-	-	-	-	-	-	-	-	-
MM#2	+	+	+	-	+	-	+	bi- allelic	-	-	-	-	-	-	c.166 C > T	p.Gln56*	1	98	+
MM#3	+	-	+	-	-	-	-	-	-	-	-	-	-	-	-	-	-	-	-
MM#4	-	+	+	-	-	-	-	-	-	-	-	-	-	-	-	-	-	-	-
MM#5	+	-	+	-	-	-	-	-	-	-	-	-	-	-	-	-	-	-	-
MM#6	-	+	+	-	-	-	-	-	-	-	-	-	-	-	-	-	-	-	-
MM#7a	-	-	+	-	-	-	+	-	-	-	-	-	-	bi- allelic (sub- clonal)	c.436 G > T	p.Glu146*	1	24	-
MM#7b	-	+	+	-	+	-	+	bi- allelic	-	-	-	-	-	mono- allelic	c.391_392dup	p.Ile132Alafs*18	1	66	-
MM#7c	+	+	+	-	+	-	+	bi- allelic	-	-	-	-	-	bi- allelic	-	-	-	-	-
MM#8	+	+	+	-	-	-	+	-	-	-	-	-	-	bi- allelic	-	-	-	-	-
MM#9	+	-	+	+	-	-	-	-	-	-	-	-	+	-	-	-	-	-	-
MM#10	+	-	+	-	+	+	-	mono- allelic	c.131-1 G > A	p. splice site mutation	2	91	-	mono- allelic	-	-	-	-	-
MM#11a	-	+	-	-	+	-	-	bi- allelic	-	-	-	-	-	-	-	-	–	-	-
MM#11b	-	+	+	-	+	-	+	bi- allelic	-	-	-	-	-	mono- allelic	c.36 T > A c.378 G > A c.597 G > A	p.Tyr12* p.Trp126* p.Trp199*	1 1 1	6 9 5	-
MM#12	-	+	+	-	+	-	-	bi- allelic	-	-	-	-	-	-	-	-	-	-	-
MM#13	-	+	+	-	-	-	-	-	-	-	-	-	-	-	-	-	-	-	-
MM#14	-	+	+	-	+	-	+	bi- allelic	-	-	-	-	-	mono- allelic	c.278_286del	p.Tyr93_Leu95del	1	79	-
MM#15a	+	-	-	-	-	-	-	-	-	-	-	-	-	-	-	-	-	-	-
MM#15b	+	-	+	-	-	+	-	-	-	-	-	-	-	mono- allelic	-	-	-	-	-
MM#16	-	+	+	-	+	-	+	mono- allelic	c.80 G > C	p.Arg27Pro	1	92	-	-	c.715 G > A c.710 G > A	p.Asp239Asn p.Trp237*	1 1	44 37	-
MM#17	-	+	+	-	-	-	-	-	-	-	-	-	-	-*	-	-	-	-	-

*biallelic deletion detected via scCNV-Seq [16].

In patient MM#16, focal *TNFRSF17* loss co-occurred with a missense mutation (p.Arg27Pro) affecting the extracellular domain of BCMA [16]. This mutation, as previously shown, impairs the binding of teclistamab and elranatamab but not alnuctamab [16]. In patient MM#10, monosomy 16 was accompanied by a splice-site mutation (c.131-1G>A, VAF 91%) in the second allele. However, in most patients with biallelic *TNFRSF17* aberrations (*n* = 5), clonal deletions resulted in complete gene loss.

In contrast, *GPRC5D* inactivation was mediated by a variety of mechanisms and often involved complex subclonal structures. Patient MM#2 with CN-LOH of 12p harbored a nonsense mutation in exon 1 (p.Gln56*, VAF 98%). Patient MM#7 carried a focal 15 kb *GPRC5D* deletion and a frameshift mutation (p.Ile132Alafs*18*)*. Patient MM#11 also carried a 12p deletion together with three low-frequency *GPRC5D* mutations (p.Tyr12*, VAF 6%; p.Trp126*, 9%; p.Trp199*, 5%). In MM#14, a large 12p deletion co-occurred with an in-frame deletion in *GPRC5D* (p.Tyr93_Leu95del). Finally, MM#16 showed compound heterozygosity with a missense mutation (p.Asp239Asn) in one allele and a nonsense mutation (p.Trp237*) in the other. Although limited in number, it is noteworthy that all double-hit *GPRC5D* lesions were detected in bone marrow biopsies, but none in those with EMD samples only.

Taken together, nearly half of the patients (8/17) exhibited biallelic antigen disruption via genomic mechanisms: predominantly homozygous deletions for *TNFRSF17*, but more diverse and subclonal alterations for *GPRC5D*. Importantly, almost one-third of patients (5/17) acquired biallelic aberrations of both BCMA and GPRC5D, underscoring immunotherapies as a major evolutionary bottleneck.

### Linear versus branching evolution

**Fig. 4:**
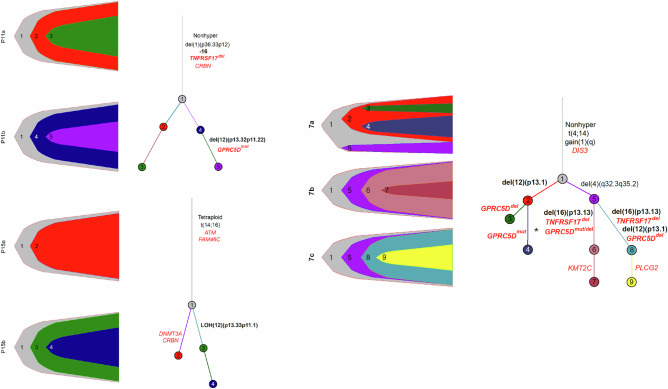
Phylogenetic clonal trees for patients MM#7, MM#11 and MM#15. Subclones are derived from the common ancestor according to their respective mutation changes detected via WGS. The lengths of the branches do not correspond to the number of mutations. *WGS revealed two additional low-frequency *GPRC5D* mutations, which were classified as tail mutations by MOBSTER and therefore not displayed in this figure.

The emergence of double- or triple-negative status for BCMA, GPRC5D, and CD38 after immunotherapies suggests a model of resistance driven by the successive accumulation of lesions in a linear fashion. However, phylogenetic trees reconstructed from longitudinal samples of three patients showed branching rather than linear evolution, consistent with initial deep responses followed by divergent clonal escape (Fig. 4). Patient MM#11, treated with AMG420 targeting BCMA (before time point 1) and talquetamab targeting GPRC5D (between time points 1 and 2), exhibited biallelic *TNFRSF17* loss in the trunk of the phylogenetic tree and a *GPRC5D* mutation in the branch dominating at time point 2. Patient MM#15, treated with ide-cel (before time point 1) and talquetamab (between time points 1 and 2), showed selection of a subclone with monoallelic *GPRC5D* loss at the second time point. Pronounced parallel evolution, with four distinct biallelic *GPRC5D* events and two independent biallelic *TNFRSF17* deletions in three dominant subclones across the three investigated time points, was observed in patient MM#7, who was treated sequentially with teclistamab, talquetamab and CAR-T. These results highlight the extraordinary adaptive capacity of late-stage myeloma.

Taken together, our sequential analyses show that progression to hepta-refractory MM arises through branching evolution across multiple coexisting subclones, rather than through a single linear trajectory dominated by one subclone.

### Comparison of WGS and IHC for antigen assessment

WGS can identify biallelic deletions of target genes, resulting in complete loss of expression. However, expression changes may also result from epigenetic remodeling or post-transcriptional regulation. To assess BCMA at the protein level, we performed immunohistochemical staining for BCMA in 12 patients with hepta-refractory MM (Supplementary Table 5).

In all 5 patients with biallelic *TNFRSF17* alterations, IHC confirmed complete loss of BCMA protein. Notably, BCMA showed no membranous staining in one patient with wild-type *TNFRSF17* and was weakly expressed (on the cell membrane) in two others. Moderate or strong staining intensities were observed in one patient each.

In summary, IHC revealed reduced or absent BCMA expression in more patients than predicted by WGS, highlighting the importance of complementary approaches and suggesting additional, as yet unidentified, resistance mechanisms.

### Whole genome sequencing to guide clinical decision making

Finally, we asked whether molecular information could, in principle, be translated into clinical practice to guide treatment decisions, such as recommending specific retreatments. To explore this retrospectively, we examined response and PFS after bispecific antibodies and CAR T-cells in relation to target antigen status as determined by WGS.

The “BCMA-after-BCMA” scenario was evaluable in 6 patients with WGS data available before a second exposure to BCMA-directed therapy. Four patients (MM#4, MM#6, MM#8, MM#15), previously treated with ide-cel (MM#15), teclistamab (MM#4, MM#6), or AMG420 (MM#8) [33], showed no *TNFRSF17* alterations by WGS and exhibited at least weak expression of BCMA by IHC. All responded to BCMA retreatment (cilta-cel = 3, ide-cel = 1) (Supplementary Table 5). Median PFS in this group was 8.6 months, and two patients remain in remission at the time of data cut-off.

By contrast, two patients (MM#2 and MM#7) harbored biallelic *TNFRSF17* deletions, with IHC confirming BCMA protein loss. Both were refractory to subsequent salvage therapies with ide-cel or cilta-cel.

Although case numbers are small, these clinical courses suggest that WGS-informed antigen profiling could help identify patients who may still benefit from retreatment, with some achieving meaningful and durable disease stabilization even in the hepta-refractory setting (Fig. 1).

## Discussion

Despite considerable advances in MM therapy, most patients will eventually exhaust all available treatment options. Building on the recently published concept of penta-refractory disease [1], we propose “hepta-refractory MM” as a new end-stage category. These patients are expected to become increasingly common where novel agents are available, representing a “new normal” in the clinic. With a median overall survival of only 1 year in this study, their prognosis remains dismal. Identifying effective therapies for hepta-refractory MM is therefore an urgent and unmet medical need.

Acknowledging the limited number of cases, our WGS analyses revealed a striking enrichment of double-hit events in tumor suppressor genes, exceeding levels previously reported [13, 32, 34–36] and accumulation of high-risk cytogenetic abnormalities [10, 37, 38], consistent with highly proliferative, apoptosis-resistant disease. Moreover, multiple genomic alterations potentially associated with resistance to both conventional and immunotherapeutic agents were identified. Notably, CRBN-pathway alterations were observed in up to 71% of patients, a frequency at least twice as high as in prior reports in the RRMM setting [14, 39]. In the future, next-generation cereblon E3 ligase modulators may still show activity in some of these cases [40]. But even when the direct activity of IMiDs in myeloma cells is impaired, IMiDs can still modulate the immune system, activating NK and T cells and exerting synergy with other myeloma drugs [41–43]. Thus, the presence of a *CRBN* mutation alone may not preclude using these agents.

A novel and clinically important finding is the loss of at least one immunotherapeutic target in roughly half of hepta-refractory patients with available WGS data, with nearly one-third losing both BCMA and GPRC5D. These losses limit further immunotherapeutic retreatment. They were predominantly mediated by biallelic events in the genes encoding BCMA, GPRC5D, or CD38, with branching evolutionary pathways evident. Importantly, the occurrence of such tumor evolution in patients who were in deep remission underscores that a hidden reservoir of persisting tumor cells drives relapse. Divergent evolution, with multiple subclones independently acquiring distinct *GPRC5D* lesions and the emergence of new *TNFRSF17* variants at later relapses, further supports the notion that more than a single myeloma cell can pass through the evolutionary bottleneck imposed by novel immunotherapies. However, two open questions arise: first, whether antigen-positive clones can reemerge over time through branching pathways, as has been described for CD20 following bispecific antibody therapy in lymphoma [44]; and second, whether combination strategies might reduce the rate of antigen loss by making simultaneous escape from multiple mechanistically distinct agents more difficult. In this context, it is notable that the majority of hepta-refractory patients with biallelic *TNFRSF17* aberrations had prior exposure to BCMA-directed TCEs, commonly administered as continuous monotherapies, whereas in our prior study of patients relapsing after intensive, multi-phase “total therapies”, IMiD-containing seven-drug combinations, we did not observe a single *CRBN* mutation [13].

It is remarkable how prior therapies leave detectable traces in the genomes of myeloma cells, allowing the impact of melphalan, IMiDs, and targeted immunotherapies to be reconstructed through WGS. These findings strongly support a central role of tumor-intrinsic resistance mechanisms in the evolution toward hepta-refractory states, and even call into question the contribution of the microenvironment [45–48] in mediating resistance, at least in a substantial fraction of patients. At the same time, nearly half of the patients progressed with wild-type antigen status, pointing to additional resistance mechanisms not explored here, potentially including microenvironmental and epigenetic mechanisms. Tumor-extrinsic mechanisms may prove more tractable, potentially mitigated by strategies such as prior debulking, γ-secretase inhibition and other methods for modulation of target antigen levels, dose optimization, or adjusted effector-to-target ratios [49–52]. Notably, in our series, CAR T-cell retreatment achieved responses in patients with preserved BCMA.

In this regard, it is encouraging that IHC consistently confirmed loss of BCMA protein in patients with double-hit *TNFRSF17* deletions. This is clinically relevant, as IHC is more widely available than WGS and can be applied even when WGS is not feasible, for example, due to a lack of sorted plasma cells. Our cohort did not include cases of BCMA retreatment in IHC-negative but WGS-intact samples. Importantly, we show that both WGS and IHC could be useful to avoid ineffective and costly therapies with potentially serious side effects in patients lacking target antigens.

Finally, the observation that some patients achieve durable survival plateaus with highly heterogeneous treatments underscores the need for personalized therapeutic strategies and refined diagnostics in this ultra-refractory population. In this context, WGS emerged as a valuable tool to guide considerations of retreatment with the same target antigen.

## Supplementary information

Supplementary material and methods

## Data Availability

Raw data are available upon request from the corresponding author.

## References

[1] Gandhi UH, Cornell RF, Lakshman A, Gahvari ZJ, McGehee E, Jagosky MH, et al. Outcomes of patients with multiple myeloma refractory to CD38-targeted monoclonal antibody therapy. Leukemia. 2019;33:2266–75. 30858549 10.1038/s41375-019-0435-7 PMC6820050

[2] Hansen DK, Sidana S, Peres LC, Colin Leitzinger C, Shune L, Shrewsbury A, et al. Idecabtagene vicleucel for relapsed/refractory multiple myeloma: real-world experience from the myeloma CAR T consortium. J Clin Oncol. 2023;41:2087–97. 36623248 10.1200/JCO.22.01365 PMC10082273

[3] Lesokhin AM, Tomasson MH, Arnulf B, Bahlis NJ, Miles Prince H, Niesvizky R, et al. Elranatamab in relapsed or refractory multiple myeloma: phase 2 MagnetisMM-3 trial results. Nat Med. 2023;29:2259–67. 37582952 10.1038/s41591-023-02528-9 PMC10504075

[4] Munshi NC, Anderson LD Jr, Shah N, Madduri D, Berdeja J, et al. Idecabtagene vicleucel in relapsed and refractory multiple myeloma. N Engl J Med. 2021;384:705–16. 33626253 10.1056/NEJMoa2024850

[5] Martin T, Usmani SZ, Berdeja JG, Agha M, Cohen AD, Hari P, et al. Ciltacabtagene autoleucel, an anti-B-cell maturation antigen chimeric antigen receptor T-cell therapy, for relapsed/refractory multiple myeloma: CARTITUDE-1 2-year follow-up. J Clin Oncol. 2023;41:1265–74. 35658469 10.1200/JCO.22.00842 PMC9937098

[6] Bahlis NJ, Costello CL, Raje NS, Levy MY, Dholaria B, Solh M, et al. Elranatamab in relapsed or refractory multiple myeloma: the MagnetisMM-1 phase 1 trial. Nat Med. 2023;29:2570–6. 37783970 10.1038/s41591-023-02589-w PMC10579053

[7] Chari A, Minnema MC, Berdeja JG, Oriol A, van de Donk NWCJ, Rodríguez-Otero P, et al. Talquetamab, a T-Cell-redirecting GPRC5D bispecific antibody for multiple myeloma. N Engl J Med. 2022;387:2232–44. 36507686 10.1056/NEJMoa2204591

[8] Moreau P, Garfall AL, van de Donk NWCJ, Nahi H, San-Miguel JF, Oriol A, et al. Teclistamab in relapsed or refractory multiple myeloma. N Engl J Med. 2022;387:495–505. 35661166 10.1056/NEJMoa2203478 PMC10587778

[9] Rasche L, Schinke C, Maura F, Bauer MA, Ashby C, Deshpande S, et al. The spatio-temporal evolution of multiple myeloma from baseline to relapse-refractory states. Nat Commun. 2022;13:4517. 35922426 10.1038/s41467-022-32145-y PMC9349320

[10] Lannes R, Samur M, Perrot A, Mazzotti C, Divoux M, Cazaubiel T, et al. In multiple myeloma, high-risk secondary genetic events observed at relapse are present from diagnosis in tiny, undetectable subclonal populations. J Clin Oncol. 2023;41:1695–702. 36343306 10.1200/JCO.21.01987 PMC10043564

[11] Keats JJ, Chesi M, Egan JB, Garbitt VM, Palmer SE, Braggio E, et al. Clonal competition with alternating dominance in multiple myeloma. Blood. 2012;120:1067–76. 22498740 10.1182/blood-2012-01-405985 PMC3412330

[12] Egan JB, Shi C-X, Tembe W, Christoforides A, Kurdoglu A, Sinari S, et al. Whole-genome sequencing of multiple myeloma from diagnosis to plasma cell leukemia reveals genomic initiating events, evolution, and clonal tides. Blood. 2012;120:1060–6. 22529291 10.1182/blood-2012-01-405977 PMC3412329

[13] Weinhold N, Ashby C, Rasche L, Chavan SS, Stein C, Stephens OW, et al. Clonal selection and double-hit events involving tumor suppressor genes underlie relapse in myeloma. Blood. 2016;128:1735–44. 27516441 10.1182/blood-2016-06-723007 PMC5043128

[14] Kortüm KM, Mai EK, Hanafiah NH, Shi C-X, Zhu Y-X, Bruins L, et al. Targeted sequencing of refractory myeloma reveals a high incidence of mutations in CRBN and Ras pathway genes. Blood. 2016;128:1226–33. 27458004 10.1182/blood-2016-02-698092 PMC5524534

[15] Samur MK, Fulciniti M, Aktas Samur A, Bazarbachi AH, Tai Y-T, Prabhala R, et al. Biallelic loss of BCMA as a resistance mechanism to CAR T cell therapy in a patient with multiple myeloma. Nat Commun. 2021;12:868. 33558511 10.1038/s41467-021-21177-5 PMC7870932

[16] Lee H, Ahn S, Maity R, Leblay N, Ziccheddu B, Truger M, et al. Mechanisms of antigen escape from BCMA- or GPRC5D-targeted immunotherapies in multiple myeloma. Nat Med. 2023;29:2295–306. 37653344 10.1038/s41591-023-02491-5 PMC10504087

[17] Da Vià MC, Dietrich O, Truger M, Arampatzi P, Duell J, Heidemeier A, et al. Homozygous BCMA gene deletion in response to anti-BCMA CAR T cells in a patient with multiple myeloma. Nat Med. 2021;27:616–9. 33619368 10.1038/s41591-021-01245-5

[18] Truger MS, Duell J, Zhou X, Heimeshoff L, Ruckdeschel A, John M, et al. Single- and double-hit events in genes encoding immune targets before and after T cell-engaging antibody therapy in MM. Blood Adv. 2021;5:3794–8. 34471932 10.1182/bloodadvances.2021004418 PMC8679680

[19] Firestone RS, Socci ND, Shekarkhand T, Zhu M, Qin WG, Hultcrantz M, et al. Antigen escape as a shared mechanism of resistance to BCMA-directed therapies in multiple myeloma. Blood. 2024;144:402–7. 38728378 10.1182/blood.2023023557 PMC11302451

[20] Derrien J, Gastineau S, Frigout A, Giordano N, Cherkaoui M, Gaborit V, et al. Acquired resistance to a GPRC5D-directed T-cell engager in multiple myeloma is mediated by genetic or epigenetic target inactivation. Nat Cancer. 2023;4:1536–43. 37653140 10.1038/s43018-023-00625-9

[21] Kumar S, Paiva B, Anderson KC, Durie B, Landgren O, Moreau P, et al. International Myeloma Working Group consensus criteria for response and minimal residual disease assessment in multiple myeloma. Lancet Oncol. 2016;17:e328–e346. 27511158 10.1016/S1470-2045(16)30206-6

[22] Rajkumar SV, Richardson P, San Miguel JF. Guidelines for the determination of the number of prior lines of therapy in multiple myeloma. Blood. 2015;126:921–2. 26272048 10.1182/blood-2015-05-647636

[23] Avet-Loiseau H, Davies FE, Samur MK, Corre J, D’Agostino M, Kaiser MF, et al. International Myeloma Society/International Myeloma Working Group consensus recommendations on the definition of high-risk multiple myeloma. J Clin Oncol. 2025;43:2553. 40561387 10.1200/JCO-25-01367 PMC13470823

[24] Fantini D, Vidimar V, Yu Y, Condello S, Meeks JJ. MutSignatures: an R package for extraction and analysis of cancer mutational signatures. Sci Rep. 2020;10:18217. 33106540 10.1038/s41598-020-75062-0 PMC7589488

[25] Rustad EH, Nadeu F, Angelopoulos N, Ziccheddu B, Bolli N, Puente XS, et al. mmsig: a fitting approach to accurately identify somatic mutational signatures in hematological malignancies. Commun Biol. 2021;4:424. 33782531 10.1038/s42003-021-01938-0 PMC8007623

[26] Caravagna G, Heide T, Williams MJ, Zapata L, Nichol D, Chkhaidze K, et al. Subclonal reconstruction of tumors by using machine learning and population genetics. Nat Genet. 2020;52:898–907. 32879509 10.1038/s41588-020-0675-5 PMC7610388

[27] Anderson KC, Kyle RA, Rajkumar SV, Stewart AK, Weber D, Richardson P, et al. Clinically relevant end points and new drug approvals for myeloma. Leukemia. 2008;22:231–9. 17972944 10.1038/sj.leu.2405016

[28] Schavgoulidze A, Talbot A, Perrot A, Cazaubiel T, Leleu X, Manier S, et al. Biallelic deletion of 1p32 defines ultra-high-risk myeloma, but monoallelic del(1p32) remains a strong prognostic factor. Blood. 2023;141:1308–15. 36375118 10.1182/blood.2022017863 PMC10163308

[29] Allmeroth K, Horn M, Kroef V, Miethe S, Müller R-U, Denzel MS. Bortezomib resistance mutations in PSMB5 determine response to second-generation proteasome inhibitors in multiple myeloma. Leukemia. 2021;35:887–92. 32690882 10.1038/s41375-020-0989-4 PMC7932915

[30] Haertle L, Barrio S, Munawar U, Han S, Zhou X, Simicek M, et al. Single-nucleotide variants and epimutations induce proteasome inhibitor resistance in multiple myeloma. Clin Cancer Res. 2023;29:279–88. 36282272 10.1158/1078-0432.CCR-22-1161

[31] Haertle L, Buenache N, Cuesta Hernández HN, Simicek M, Snaurova R, Rapado I et al. Genetic alterations in members of the proteasome 26S subunit, AAA-ATPase () gene family in the light of proteasome inhibitor resistance in multiple myeloma. Cancers 2023;15. 10.3390/cancers15020532. 10.3390/cancers15020532 PMC9856285 36672481

[32] Giesen N, Paramasivam N, Toprak UH, Huebschmann D, Xu J, Uhrig S, et al. Comprehensive genomic analysis of refractory multiple myeloma reveals a complex mutational landscape associated with drug resistance and novel therapeutic vulnerabilities. Haematologica. 2022;107:1891–901. 35045690 10.3324/haematol.2021.279360 PMC9335090

[33] Topp MS, Duell J, Zugmaier G, Attal M, Moreau P, Langer C, et al. Anti-B-cell maturation antigen BiTE molecule AMG 420 induces responses in multiple myeloma. J Clin Oncol. 2020;38:775–83. 31895611 10.1200/JCO.19.02657

[34] Liu E, Sudha P, Becker N, Jaouadi O, Suvannasankha A, Lee K, et al. Identifying novel mechanisms of biallelic TP53 loss refines poor outcome for patients with multiple myeloma. Blood Cancer J. 2023;13:144. 37696786 10.1038/s41408-023-00919-2 PMC10495448

[35] Ansari-Pour N, Samur M, Flynt E, Gooding S, Towfic F, Stong N, et al. Whole-genome analysis identifies novel drivers and high-risk double-hit events in relapsed/refractory myeloma. Blood. 2023;141:620–33. 36223594 10.1182/blood.2022017010 PMC10163277

[36] Chavan SS, He J, Tytarenko R, Deshpande S, Patel P, Bailey M, et al. Bi-allelic inactivation is more prevalent at relapse in multiple myeloma, identifying RB1 as an independent prognostic marker. Blood Cancer J. 2017;7:e535. 28234347 10.1038/bcj.2017.12 PMC5386330

[37] Yan Y, Qin X, Liu J, Fan H, Yan W, Liu L, et al. Clonal phylogeny and evolution of critical cytogenetic aberrations in multiple myeloma at the single-cell level by QM-FISH. Blood Adv. 2022;6:441–51. 34653241 10.1182/bloodadvances.2021004992 PMC8791565

[38] Merz M, Jauch A, Hielscher T, Mai EK, Seckinger A, Hose D, et al. Longitudinal fluorescence hybridization reveals cytogenetic evolution in myeloma relapsing after autologous transplantation. Haematologica. 2017;102:1432–8. 28495913 10.3324/haematol.2017.168005 PMC5541876

[39] Gooding S, Ansari-Pour N, Towfic F, Ortiz Estévez M, Chamberlain PP, Tsai K-T, et al. Multiple cereblon genetic changes are associated with acquired resistance to lenalidomide or pomalidomide in multiple myeloma. Blood. 2021;137:232–7. 33443552 10.1182/blood.2020007081 PMC7893409

[40] Bird S, Pawlyn C. IMiD resistance in multiple myeloma: current understanding of the underpinning biology and clinical impact. Blood. 2023;142:131–40. 36929172 10.1182/blood.2023019637

[41] D’Souza A, Costa LJ, San-Miguel JF, Berdeja JG, Morillo Giles D, Touzeau C, et al. Teclistamab, daratumumab, and pomalidomide in patients with relapsed/refractory multiple myeloma: results from the MajesTEC-2 cohort and trimm-2 studies. Blood. 2024;144:495.

[42] Danhof S, Schreder M, Knop S, Rasche L, Strifler S, Löffler C, et al. Expression of programmed death-1 on lymphocytes in myeloma patients is lowered during lenalidomide maintenance. Haematologica. 2018;103:e126–e129. 29191843 10.3324/haematol.2017.178947 PMC5830376

[43] Davies FE, Raje N, Hideshima T, Lentzsch S, Young G, Tai YT, et al. Thalidomide and immunomodulatory derivatives augment natural killer cell cytotoxicity in multiple myeloma. Blood. 2001;98:210–6. 11418482 10.1182/blood.v98.1.210

[44] Duell J, Leipold AM, Appenzeller S, Fuhr V, Rauert-Wunderlich H, Da Via M, et al. Sequential antigen loss and branching evolution in lymphoma after CD19- and CD20-targeted T-cell-redirecting therapy. Blood. 2024;143:685–96. 37976456 10.1182/blood.2023021672 PMC10900140

[45] Iannozzi NT, Giuliani N, Storti P. Deciphering the bone marrow microenvironment’s role in multiple myeloma immunotherapy resistance. Front Immunol. 2025;16:1613265. 40755766 10.3389/fimmu.2025.1613265 PMC12313686

[46] Tirier SM, Mallm J-P, Steiger S, Poos AM, Awwad MHS, Giesen N, et al. Subclone-specific microenvironmental impact and drug response in refractory multiple myeloma revealed by single-cell transcriptomics. Nat Commun. 2021;12:6960. 34845188 10.1038/s41467-021-26951-z PMC8630108

[47] John M, Helal M, Duell J, Mattavelli G, Stanojkovska E, Afrin N, et al. Spatial transcriptomics reveals profound subclonal heterogeneity and T-cell dysfunction in extramedullary myeloma. Blood. 2024;144:2121–35. 39172759 10.1182/blood.2024024590

[48] Bhowmick K, von Suskil M, Al-Odat OS, Elbezanti WO, Jonnalagadda SC, Budak-Alpdogan T, et al. Pathways to therapy resistance: the sheltering effect of the bone marrow microenvironment to multiple myeloma cells. Heliyon. 2024;10:e33091. 39021902 10.1016/j.heliyon.2024.e33091 PMC11252793

[49] Waldschmidt JM, Rasche L. BCMA bispecifics: breaking the chains of resistance. Blood. 2024;144:2566–8. 39699921 10.1182/blood.2024026932

[50] Lee H, Durante M, Skerget S, Vishwamitra D, Benaoudia S, Ahn S, et al. Impact of soluble BCMA and non-T-cell factors on refractoriness to BCMA-targeting T-cell engagers in multiple myeloma. Blood. 2024;144:2637–51. 39321344 10.1182/blood.2024026212 PMC11738017

[51] Rieger L, Irlinger K, Füchsl F, Tietje M, Purcarea A, Barbian NM et al. Boosting CAR T-cell efficacy by blocking proteasomal degradation of membrane antigens. Blood 2025. 10.1182/blood.2024027616. 10.1182/blood.2024027616 PMC12883852 40638685

[52] Neubert S, Munawar U, Mersi J, Noderer J, Nerreter S, Kurian S, et al. Plasmapheresis facilitates soluble BCMA clearance and contributes to reversing primary resistance to anti-BCMA immunotherapy in multiple myeloma. Leukemia. 2025;39:2563–7. 40931044 10.1038/s41375-025-02757-6 PMC12463651

