# Supplementary material for: The evolution to hepta-refractory myeloma involves sequential loss of CD38, BCMA and GPRC5D
Source: Leukemia. 2026 Feb 17;40(4):730–8. doi: 10.1038/s41375-026-02889-3 (PMC13056581; doi:10.1038/s41375-026-02889-3)
Supplement: Supplementary file 1 — Supplementary material and methods [file 41375_2026_2889_MOESM1_ESM.pdf]

Supplemental Material and Methods

Supplemental Table 1: Genes analyzed by WGS for SNV

ABCB1	*CBS*	*DIS3*	*GPRC5D*	*KMT2B*	*NR3C1*	*PSMD14*	*SLAMF7*
ABCB4	*CCND1*	*DNMT3A*	*HIF1A*	*KRAS*	*NRAS*	*PSMD5*	*SLC35G5*
ABCG2	*CD19*	*DRG1*	*HSPA5*	*MAF*	*PCLO*	*PSMD6*	*SLC35G6*
ABL1	*CD38*	*DUOX2*	*HUWE1*	*MAFB*	*PDGFRA*	*PSMG2*	*SP140*
ATM	*CDKN1B*	*DUSP2*	*IDH1*	*MAML2*	*PIGO*	*PTPN11*	*STAT3*
ATR	*CDKN2C*	*EGFR*	*IDH2*	*MAP2K1*	*PIK3CA*	*RARA*	*TET2*
B2M	*CKS1B*	*EGR1*	*IGF1R*	*MAX*	*PIM1*	*RASA2*	*TLR4*
BDNF	*COPS3*	*EPHB2*	*IKZF1*	*MET*	*PIM3*	*RB1*	*TNFRSF17*
BIRC2	*COPS4*	*ERBB2*	*IKZF3*	*MPL*	*PKNOX1*	*RBX1*	*TP53*
BIRC3	*COPS8*	*ERBB4*	*IKZF4*	*MUC16*	*PRDM1*	*RET*	*TRAF2*
BRAF	*CRBN*	*EZH2*	*IL6ST*	*MUC2*	*PSMB5*	*RIPK4*	*TRAF3*
BRCA1	*CUL4A*	*FAM46C*	*IRF4*	*MYC*	*PSMB8*	*ROBO2*	*TTN*
BRCA2	*CUL4B*	*FGFR2*	*JAK2*	*MYCBP2*	*PSMC2*	*ROS1*	*UBE2G1*
BSG	*CXCR4*	*FGFR3*	*KDM6A*	*MYD88*	*PSMC5*	*RPS3A*	*WHSC1*
BTG1	*CYLD*	*FGFR4*	*KIF5B*	*NEDD8*	*PSMC6*	*RRBP1*	*XBP1*
CAND1	*DDB1*	*FLT3*	*KIT*	*NFKB2*	*PSMD1*	*SF3B1*	*XPO1*
CARD11	*DDR2*	*FLT3-ITD*	*KMT2A*	*NFKBIA*	*PSMD10*	*SHC1*	

Supplemental Table 2: Clinical characteristics of patients with hepta-refractory MM

**Characteristic (at occurrence of hepta-refractoriness)**	**Result**
Median age - yr (range)	62.0 (37-85)
Gender: female/male – no.(%)	13 (35.1%) / 24 (64.9%)
Median time since diagnosis - yr (range)	7.6 (2.0 - 20.1)
Extramedullary disease - no./total no. (%)	20/32 (62.5%)
% Plasma cells in bone marrow - median, data availability no./total no.	50%, 13/37
ISS 1/2/3 - no./total no. (%)	12/32 (37.5%)<br>8/32 (25.0%)<br>12/32 (37.5%)
High risk cytogenetic profile - no./total no. (%)	22/34 (64.7%)
Median number of previous lines of therapy (range)	9.0 (5-17)
(First) BCMA-directed immunotherapy<br>Ciltacabtagene-autoleucel (cilta-cel)- no./total no. (%)<br>Idecabtagene-lecleucel (ide-cel) - no./total no. (%)<br>TCE - no./total no. (%)	 2/37 (5.4%)<br>14/37 (37.8%)<br>21/37 (56.8%)
Penta-refractory disease at start of first BCMA-/GPRC5D-directed therapy - no./total no. (%)	25/37 (67.6%)
Lines of therapy after occurrence of hepta-refractoriness<br>no subsequent therapy - no./total no. (%)<br>≥ 1 subsequent line of therapy - no./total no. (%)<br>≥ 2 subsequent lines of therapy - no./total no. (%)<br>≥ 3 subsequent lines of therapy - no./total no. (%)	 4/37 (10.8%)<br>33/37 (89.2%)<br>18/37 (48.6%)<br>11/37 (29.7%)

Supplemental Table 3: Administered treatments in hepta-refractory disease

**First line of therapy in hepta-refractoriness**	**No./total no. (%)**
Immunotherapy with TCE/CAR T-cells	6/33 (18.2%)
Selinexor-based	5/33 (15.2%)
Polychemotherapy or high-dose melphalan	10/33 (30.3%)
PI-/IMiD-/CD38-based therapy	9/33 (27.3%)
Other (e.g. Venetoclax, …)	3/33 (9.1%)
**Second line of therapy in hepta-refractoriness**	
Immunotherapy with TCE/CAR T-cells	8/18 (44.4%)
Selinexor-based	2/18 (11.1%)
Polychemotherapy or high-dose melphalan	4/18 (22.2%)
PI-/IMiD-/CD38-based therapy	3/18 (16.7%)
Other (e.g. Venetoclax, …)	1/18 (5.6%)
**Third line of therapy in hepta-refractoriness**	
Immunotherapy with TCE/CAR T-cells	3/11 (27.3%)
Selinexor-based	3/11 (27.3%)
Polychemotherapy or high-dose melphalan	2/11 (18.2%)
PI-/IMiD-/CD38-based therapy	1/11 (9.1%)
Other (e.g. Venetoclax, …)	2/11 (18.2%)

Supplemental Table 4: SNV detected by WGS

Pat ID	Sample site	symbol	HGVSc	HGVSp	vaf	ensg	bio-type	conse-quence	exon	intron	result
MM#5	BM	ABCB4	ENST00000265723.4:c.1054C>A	ENSP00000265723.4:p.Pro352Thr	0.53	ENSG00000005471	protein coding	missense variant	(10/28)		variant
MM#1	EMD	ATM	ENST00000278616.4:c.7343A>T	ENSP00000278616.4:p.Asp2448Val	0.41	ENSG00000149311	protein coding	missense variant	(50/63)		variant
MM#15a	BM	ATM	ENST00000278616.4:c.8662A>T	ENSP00000278616.4:p.Ile2888Leu	0.37	ENSG00000149311	protein coding	missense variant	(59/63)		variant
MM#15b	BM	ATM	ENST00000278616.4:c.8662A>T	ENSP00000278616.4:p.Ile2888Leu	0.38	ENSG00000149311	protein coding	missense variant	(59/63)		variant
MM#16	BM	ATM	ENST00000278616.4:c.205C>T	ENSP00000278616.4:p.Gln69*	0.43	ENSG00000149311	protein coding	stop gained	(4/63)		positive
MM#16	BM	ATM	ENST00000278616.4:c.7330G>A	ENSP00000278616.4:p.Glu2444Lys	0.45	ENSG00000149311	protein coding	missense variant	(50/63)		positive T2
MM#6	EMD	BRAF	ENST00000288602.6:c.1790T>G	ENSP00000288602.6:p.Leu597Arg	0.48	ENSG00000157764	protein coding	missense variant	(15/18)		positive
MM#6	EMD	CD19	ENST00000538922.1:c.183_185 del	ENSP00000437940.1:p.Phe61del	0.56	ENSG00000177455	protein coding	inframe deletion	(2/15)		variant
MM#10	EMD	CDKN2C	ENST00000262662.1:c.445del	ENSP00000262662.1:p.Arg149Glyfs Ter7	0.91	ENSG00000123080	protein coding	frameshift variant	(4/4)		positive
MM#16	BM	CDKN2C	ENST00000262662.1:c.386_395 dup	ENSP00000262662.1:p.His132Glnfs Ter8	0.17	ENSG00000123080	protein coding	frameshift variant	(4/4)		positive
MM#10	EMD	COPS3	ENST00000268717.5:c.128A>T	ENSP00000268717.5:p.Asp43Val	0.85	ENSG00000141030	protein coding	missense variant	(2/12)		positive T2
MM#11a	BM	CRBN	ENST00000231948.4:c.22C>T	ENSP00000231948.4:p.Gln8*	0.23	ENSG00000113851	protein coding	stop gained	(1/11)		positive
MM#11b	BM	CRBN	ENST00000231948.4:c.22C>T	ENSP00000231948.4:p.Gln8*	0.19	ENSG00000113851	protein coding	stop gained	(1/11)		positive
MM#15a	BM	CRBN	ENST00000231948.4:c.474A>C	ENSP00000231948.4:p.Lys158Asn	0.56	ENSG00000113851	protein coding	missense variant	(4/11)		variant
MM#15b	BM	CRBN	ENST00000231948.4:c.527+2T>G		0.75	ENSG00000113851	protein coding	splice donor variant		(4/10)	positive
MM#4	EMD	CUL4B	ENST00000404115.3:c.1186C>A	ENSP00000384109.3:p.Leu396Ile	0.27	ENSG00000158290	protein coding	missense variant	(9/22)		variant
MM#7c	BM	CUL4B	ENST00000404115.3:c.857T>A	ENSP00000384109.3:p.Leu286*	0.12	ENSG00000158290	protein coding	stop gained	(6/22)		positive
MM#17	EMD	CUL4B	ENST00000404115.3:c.2233G>T	ENSP00000384109.3:p.Val745Phe	0.81	ENSG00000158290	protein coding	missense variant	(19/22)		positive T2
MM#2	PB	CYLD	ENST00000427738.3:c.1814C>T	ENSP00000392025.3:p.Ser605Leu	0.27	ENSG00000083799	protein coding	missense variant	(10/18)		variant
MM#16	BM	CYLD	ENST00000427738.3:c.2071C>T	ENSP00000392025.3:p.His691Tyr	0.49	ENSG00000083799	protein coding	missense variant	(13/18)		variant
MM#9	BM	DDB1	ENST00000301764.7:c.3253G>A	ENSP00000301764.7:p.Ala1085Thr	0.22	ENSG00000167986	protein coding	missense variant	(26/27)		variant
MM#7a	BM	DIS3	ENST00000377767.4:c.2024C>T	ENSP00000366997.4:p.Ala675Val	0.68	ENSG00000083520	protein coding	missense variant	(16/21)		variant
MM#7b	BM	DIS3	ENST00000377767.4:c.2024C>T	ENSP00000366997.4:p.Ala675Val	0.61	ENSG00000083520	protein coding	missense variant	(16/21)		variant
MM#7c	BM	DIS3	ENST00000377767.4:c.2024C>T	ENSP00000366997.4:p.Ala675Val	0.58	ENSG00000083520	protein coding	missense variant	(16/21)		variant

Pat ID	sample site	symbol	HGVSc	HGVSp	vaf	ensg	bio-type	conse-quence	exon	intron	result
MM#15a	BM	DNMT3A	ENST00000264709.3:c.2246G>A	ENSP00000264709.3:p.Arg749His	0.41	ENSG00000119772	protein coding	missense variant	(19/23)		positive T2
MM#16	BM	EGFR	ENST00000275493.2:c.3203G>A	ENSP00000275493.2:p.Arg1068Gln	0.49	ENSG00000146648	protein coding	missense variant	(27/28)		variant
MM#15b	BM	ERBB2	ENST00000406381.2:c.1346C>T	ENSP00000385185.2:p.Thr449Met	0.36	ENSG00000141736	protein coding	missense variant	(14/29)		positive T2
MM#15a	BM	FAM46C	ENST00000369448.3:c.847_848 dup	ENSP00000358458.3:p.Gln284Asnfs Ter26	0.79	ENSG00000183508	protein coding	frameshift variant	(2/2)		positive
MM#9	BM	FAM46C	ENST00000369448.3:c.123dup	ENSP00000358458.3:p.Pro42SerfsT er68	0.83	ENSG00000183508	protein coding	frameshift variant	(2/2)		positive
MM#15b	BM	FAM46C	ENST00000369448.3:c.847_848 dup	ENSP00000358458.3:p.Gln284Asnfs Ter26	0.86	ENSG00000183508	protein coding	frameshift variant	(2/2)		positive
MM#8	BM	FGFR3	ENST00000340107.4:c.742C>T	ENSP00000339824.4:p.Arg248Cys	0.09	ENSG00000068078	protein coding	missense variant splice region variant	(7/18)		positive
MM#7a	BM	GPRC5D	ENST00000228887.1:c.436G>T	ENSP00000228887.1:p.Glu146*	0.24	ENSG00000111291	protein coding	stop gained	(1/3)		positive
MM#11b	BM	GPRC5D	ENST00000228887.1:c.378G>A	ENSP00000228887.1:p.Trp126*	0.09	ENSG00000111291	protein coding	stop gained	(1/3)		positive
MM#11b	BM	GPRC5D	ENST00000228887.1:c.36T>A	ENSP00000228887.1:p.Tyr12*	0.06	ENSG00000111291	protein coding	stop gained	(1/3)		positive
MM#11b	BM	GPRC5D	ENST00000228887.1:c.597G>A	ENSP00000228887.1:p.Trp199*	0.04	ENSG00000111291	protein coding	stop gained	(1/3)		positive
MM#7b	BM	GPRC5D	ENST00000228887.1:c.391_392 dup	ENSP00000228887.1:p.Ile132Alafs Ter18	0.66	ENSG00000111291	protein coding	frameshift variant	(1/3)		positive
MM#2	PB	GPRC5D	ENST00000228887.1:c.166C>T	ENSP00000228887.1:p.Gln56*	0.98	ENSG00000111291	protein coding	stop gained	(1/3)		positive
MM#14	BM	GPRC5D	ENST00000228887.1:c.278_286 del	ENSP00000228887.1:p.Tyr93_Leu95 del	0.79	ENSG00000111291	protein coding	inframe deletion	(1/3)		positive T2
MM#16	BM	GPRC5D	ENST00000228887.1:c.715G>A	ENSP00000228887.1:p.Asp239Asn	0.44	ENSG00000111291	protein coding	missense variant	(1/3)		positive T2
MM#16	BM	GPRC5D	ENST00000228887.1:c.710G>A	ENSP00000228887.1:p.Trp237*	0.37	ENSG00000111291	protein coding	stop gained	(1/3)		positive
MM#15b	BM	HUWE1	ENST00000342160.3:c.1057G>A	ENSP00000340648.3:p.Ala353Thr	0.44	ENSG00000086758	protein coding	missense variant	(13/83)		positive T2
MM#16	BM	HUWE1	ENST00000342160.3:c.3433G>A	ENSP00000340648.3:p.Glu1145Lys	0.46	ENSG00000086758	protein coding	missense variant	(30/83)		positive T2
MM#5	BM	KDM6A	ENST00000377967.4:c.1924-1G>A		0.87	ENSG00000147050	protein coding	splice acceptor variant	(16/28)	positive	
MM#6	EMD	KIT	ENST00000288135.5:c.850G>A	ENSP00000288135.5:p.Asp284Asn	0.10	ENSG00000157404	protein coding	missense variant	(5/21)		variant
MM#1	EMD	KMT2B	ENST00000222270.7:c.2675G>A	ENSP00000222270.6:p.Arg892His	0.38	ENSG00000272333	protein coding	missense variant	(5/37)		variant
MM#3	EMD	KMT2B	ENST00000222270.7:c.6641dup	ENSP00000222270.6:p.Val2215Serf sTer88	0.38	ENSG00000272333	protein coding	frameshift variant	(28/37)		positive
MM#5	BM	KMT2B	ENST00000222270.7:c.2860C>T	ENSP00000222270.6:p.Pro954Ser	0.51	ENSG00000272333	protein coding	missense variant	(6/37)		variant
MM#13	EMD	KRAS	ENST00000256078.4:c.183A>C	ENSP00000256078.4:p.Gln61His	0.48	ENSG00000133703	protein coding	missense variant	(3/6)		positive

Pat ID	sample site	symbol	HGVSc	HGVSp	vaf	ensg	bio-type	conse-quence	exon	intron	result
MM#2	PB	KRAS	ENST00000256078.4:c.35G>C	ENSP00000256078.4:p.Gly12Ala	0.99	ENSG00000133703	protein coding	missense variant	(2/6)		positive
MM#4	EMD	KRAS	ENST00000256078.4:c.35G>A	ENSP00000256078.4:p.Gly12Asp	0.39	ENSG00000133703	protein coding	missense variant	(2/6)		positive
MM#5	BM	KRAS	ENST00000256078.4:c.540T>A	ENSP00000256078.4:p.Cys180*	0.46	ENSG00000133703	protein coding	stop gained	(5/6)		variant
MM#12	EMD	KRAS	ENST00000256078.4:c.183A>C	ENSP00000256078.4:p.Gln61His	0.40	ENSG00000133703	protein coding	missense variant	(3/6)		positive
MM#14	BM	KRAS	ENST00000256078.4:c.35G>A	ENSP00000256078.4:p.Gly12Asp	0.65	ENSG00000133703	protein coding	missense variant	(2/6)		positive
MM#16	BM	KRAS	ENST00000256078.4:c.34G>A	ENSP00000256078.4:p.Gly12Ser	0.47	ENSG00000133703	protein coding	missense variant	(2/6)		positive
MM#17	EMD	KRAS	ENST00000256078.4:c.183A>C	ENSP00000256078.4:p.Gln61His	0.39	ENSG00000133703	protein coding	missense variant	(3/6)		positive
MM#3	EMD	MAX	ENST00000358664.4:c.137T>G	ENSP00000351490.4:p.Leu46Trp	0.64	ENSG00000125952	protein coding	missense variant	(3/5)		positive T2
MM#15b	BM	MUC2	ENST00000441003.2:c.3140C>T	ENSP00000415183.2:p.Ala1047Val	0.37	ENSG00000198788	protein coding	missense variant	(23/49)		positive T2
MM#17	EMD	MUC2	ENST00000441003.2:c.2446G>C	ENSP00000415183.2:p.Val816Leu	0.38	ENSG00000198788	protein coding	missense variant	(19/49)		positive T2
MM#14	BM	MYC	ENST00000377970.2:c.82G>T	ENSP00000367207.2:p.Asp28Tyr	0.21	ENSG00000136997	protein coding	missense variant	(2/3)		positive T2
MM#16	BM	MYC	ENST00000377970.2:c.482C>T	ENSP00000367207.2:p.Ser161Leu	0.44	ENSG00000136997	protein coding	missense variant	(2/3)		positive
MM#6	EMD	NRAS	ENST00000369535.4:c.182A>G	ENSP00000358548.4:p.Gln61Arg	0.63	ENSG00000213281	protein coding	missense variant	(3/7)		positive
MM#3	EMD	NRAS	ENST00000369535.4:c.182A>G	ENSP00000358548.4:p.Gln61Arg	0.38	ENSG00000213281	protein coding	missense variant	(3/7)		positive
MM#12	EMD	NRAS	ENST00000369535.4:c.145G>A	ENSP00000358548.4:p.Glu49Lys	0.68	ENSG00000213281	protein coding	missense variant	(3/7)		variant
MM#9	BM	NRAS	ENST00000369535.4:c.37G>C	ENSP00000358548.4:p.Gly13Arg	0.74	ENSG00000213281	protein coding	missense variant	(2/7)		positive
MM#15a	BM	PCLO	ENST00000333891.9:c.15253C>T	ENSP00000334319.8:p.Arg5085*	0.41	ENSG00000186472	protein coding	stop gained	(24/25)		positive
MM#13	EMD	PCLO	ENST00000333891.9:c.1124_1125delinsCTCTTGGTCCTGCTAAGCCTCCAGCTCAGCAC	ENSP00000334319.8:p.Gln375delinsProLeuGlyProAlaLysProProAlaGlnHis	0.42	ENSG00000186472	protein coding	protein altering variant	(2/25)		variant
MM#8	BM	PCLO	ENST00000333891.9:c.12296A>T	ENSP00000334319.8:p.Gln4099Leu	0.25	ENSG00000186472	protein coding	missense variant	(7/25)		variant
MM#4	EMD	PCLO	ENST00000333891.9:c.13945C>G	ENSP00000334319.8:p.His4649Asp	0.32	ENSG00000186472	protein coding	missense variant	(13/25)		variant
MM#6	EMD	PCLO	ENST00000333891.9:c.12914T>A	ENSP00000334319.8:p.Ile4305Asn	0.53	ENSG00000186472	protein coding	missense variant	(7/25)		variant
MM#15b	BM	PCLO	ENST00000333891.9:c.15253C>T	ENSP00000334319.8:p.Arg5085*	0.43	ENSG00000186472	protein coding	stop gained	(24/25)		positive
MM#2	PB	PIK3CA	ENST00000263967.3:c.1633G>A	ENSP00000263967.3:p.Glu545Lys	0.57	ENSG00000121879	protein coding	missense variant	(10/21)		positive
MM#16	BM	PIK3CA	ENST00000263967.3:c.1633G>A	ENSP00000263967.3:p.Glu545Lys	0.34	ENSG00000121879	protein coding	missense variant	(10/21)		positive T2

Pat ID	sample site	symbol	HGVSc	HGVSp	vaf	ensg	bio-type	conse-quence	exon	intron	result
MM#3	EMD	RB1	ENST00000267163.4:c.608-9_638del		0,57	ENSG00000139687	protein coding	splice acceptor variant coding sequence variant intron variant	(7/27)	(6/26)	variant
MM#17	EMD	RET	ENST00000355710.3:c.2781C>G	ENSP00000347942.3:p.Ile927Met	0,45	ENSG00000165731	protein coding	missense variant	(16/20)		variant
MM#1	EMD	RIPK4	ENST00000332512.3:c.2212G>A	ENSP00000332454.3:p.Ala738Thr	0,40	ENSG00000183421	protein coding	missense variant	(8/8)		variant
MM#5	BM	ROBO2	ENST00000487694.3:c.1221C>A	ENSP00000417335.2:p.Cys407*	0,51	ENSG00000185008	protein coding	stop gained	(9/27)		positive
MM#8	BM	SP140	ENST00000392045.3:c.1426C>T	ENSP00000375899.3:p.Gln476*	0,15	ENSG00000079263	protein coding	stop gained	(14/27)		positive
MM#10	EMD	TNFR SF17	ENST00000053243.1:c.131-1G>A		0,91	ENSG00000048462	protein coding	splice acceptor variant	(1/2)	positive	
MM#16	BM	TNFR SF17	ENST00000053243.1:c.80G>C	ENSP00000053243.1:p.Arg27Pro	0,92	ENSG00000048462	protein coding	missense variant	(1/3)		positive T2
MM#1	EMD	TP53	ENST00000269305.4:c.843C>A	ENSP00000269305.4:p.Asp281Glu	0,70	ENSG00000141510	protein coding	missense variant	(8/11)		positive
MM#2	PB	TP53	ENST00000269305.4:c.733G>A	ENSP00000269305.4:p.Gly245Ser	0,93	ENSG00000141510	protein coding	missense variant	(7/11)		positive
MM#6	EMD	TP53	ENST00000269305.4:c.719G>T	ENSP00000269305.4:p.Ser240Ile	0,89	ENSG00000141510	protein coding	missense variant	(7/11)		positive T2
MM#3	EMD	TP53	ENST00000269305.4:c.637C>T	ENSP00000269305.4:p.Arg213*	0,69	ENSG00000141510	protein coding	stop gained	(6/11)		positive
MM#10	EMD	TP53	ENST00000269305.4:c.96+1G>T		0,86	ENSG00000141510	protein coding	splice donor variant		(3/10)	positive
MM#17	EMD	TP53	ENST00000269305.4:c.590T>G	ENSP00000269305.4:p.Val197Gly	0,70	ENSG00000141510	protein coding	missense variant	(6/11)		positive T2
MM#4	EMD	TTN	ENST00000589042.1:c.61933G>C	ENSP00000467141.1:p.Glu20645Gln	0,42	ENSG00000155657	protein coding	missense variant	(304/363)		variant
MM#3	EMD	TTN	ENST00000589042.1:c.82579C>G	ENSP00000467141.1:p.Leu27527Val	0,37	ENSG00000155657	protein coding	missense variant	(326/363)		variant
MM#3	EMD	TTN	ENST00000589042.1:c.57727G>A	ENSP00000467141.1:p.Ala19243Thr	0,41	ENSG00000155657	protein coding	missense variant	(295/363)		variant
MM#9	BM	TTN	ENST00000589042.1:c.49151_49152del	ENSP00000467141.1:p.Thr16384LysfsTer10	0,40	ENSG00000155657	protein coding	frameshift variant	(262/363)		positive
MM#16	BM	TTN	ENST00000589042.1:c.52486G>C	ENSP00000467141.1:p.Asp17496His	0,47	ENSG00000155657	protein coding	missense variant	(275/363)		positive T2
MM#6	EMD	XBP1	ENST00000216037.6:c.243_244del	ENSP00000216037.6:p.Arg81SerfsTer16	0,51	ENSG00000100219	protein coding	frameshift variant	(2/5)		positive

Supplemental Table 5: WGS and IHC results in relation to clinical outcome after BCMA-targeted re-exposure

Pat. ID	WGS finding *TNFRSF17* locus	BCMA IHC localization and intensity (score)	Proportion of BCMA-positive plasma cells (%) (score)	Total score BCMA (range 0-9)	(Subsequent) BCMA re-exposure
MM#1	monoallelic deletion	negative (0)	0% (0)	0	elranatamab after AMG420 (WGS/IHC after re-exposure), PFS 0.2 months
MM#2	biallelic deletion	negative (0)	0% (0)	0	ide-cel after teclistamab, PFS 1.7 months
MM#3	none	membranous: negative (0) cytoplasmatic/Golgi area (dot-like): weak-moderate intensity	0% (0)	0	no
MM#4	none	membranous: weak intensity (1) cytoplasmatic/Golgi area (dot-like): intense	100% (3)	3	cilta-cel after teclistamab, PFS 6.9 months
MM#5	none	membranous: moderate intensity (2) cytoplasmatic/Golgi area (dot-like): intense	>95% (3)	6	no
MM#6	none	membranous: intense (3) cytoplasmatic/Golgi area (dot-like): intense	100% (3)	9	ide-cel + carfilzomib consolidation after teclistamab, died at 8.6 months in remission due to septic shock
MM#7	biallelic deletion	negative (0)	0% (0)	0	cilta-cel after teclistamab, PFS 1.2 months
MM#8	none	membranous: weak intensity (1) cytoplasmatic/Golgi area (dot-like): intense	60% (1)	1	cilta-cel after AMG420, remains in remission at 24.0 months
MM#9	loss of heterozygosity	membranous: intense (3) cytoplasmatic/Golgi area (dot-like): intense	100% (3)	9	no
MM#10	biallelic hit (deletion + mutation)	negative (0)	0% (0)	0	no
MM#11	biallelic deletion	negative (0)	0% (0)	0	no
MM#24	biallelic deletion	negative (0)	0% (0)	0	no

BCMA staining was scored as negative (0), weak (1+), moderate (2+), or strong (3+), and the percentage of positively stained tumor cells was scored as 1+ (< 70% positive cells), 2+ (70-95%), and 3+ (>95%). The final score was calculated by multiplying these two variables.
